# A bioassay-guided fractionation system to identify endogenous small molecules that activate plasma membrane H^+^-ATPase activity in Arabidopsis

**DOI:** 10.1093/jxb/erx156

**Published:** 2017-06-03

**Authors:** Xiuli Han, Yongqing Yang, Yujiao Wu, Xiaohui Liu, Xiaoguang Lei, Yan Guo

**Affiliations:** 1 State Key Laboratory of Plant Physiology and Biochemistry, College of Biological Sciences, China Agricultural University, Beijing, China; 2 National Institute of Biological Sciences, Beijing, China; 3 Beijing National Laboratory for Molecular Sciences, Key Laboratory of Bioorganic Chemistry and Molecular Engineering of Ministry of Education, Department of Chemical Biology, College of Chemistry and Molecular Engineering, Synthetic and Functional Biomolecules Center, and Peking-Tsinghua Center for Life Sciences, Peking University, Beijing, China

**Keywords:** Arabidopsis, bioassay-guided fractionation, plasma membrane H^+^-ATPase, unsaturated fatty acids

## Abstract

Plasma membrane (PM) H^+^-ATPase is essential for plant growth and development. Various environmental stimuli regulate its activity, a process that involves many protein cofactors. However, whether endogenous small molecules play a role in this regulation remains unknown. Here, we describe a bio-guided isolation method to identify endogenous small molecules that regulate PM H^+^-ATPase activity. We obtained crude extracts from Arabidopsis seedlings with or without salt treatment and then purified them into fractions based on polarity and molecular mass by repeated column chromatography. By evaluating the effect of each fraction on PM H^+^-ATPase activity, we found that fractions containing the endogenous, free unsaturated fatty acids oleic acid (C18:1), linoleic acid (C18:2), and linolenic acid (C18:3) extracted from salt-treated seedlings stimulate PM H^+^-ATPase activity. These results were further confirmed by the addition of exogenous C18:1, C18:2, or C18:3 in the activity assay. The *ssi2* mutant, with reduced levels of C18:1, C18:2, and C18:3, displayed reduced PM H^+^-ATPase activity. Furthermore, C18:1, C18:2, and C18:3 directly bound to the C-terminus of the PM H^+^-ATPase AHA2. Collectively, our results demonstrate that the binding of free unsaturated fatty acids to the C-terminus of PM H^+^-ATPase is required for its activation under salt stress. The bio-guided isolation model described in this study could enable the identification of new endogenous small molecules that modulate essential protein functions, as well as signal transduction, in plants.

## Teaser

Endogenous unsaturated fatty acids identified from salt-treated Arabidopsis by a bioassay-guided fractionation method activate plasma membrane H^+^-ATPase by binding to its C-terminus.

## Introduction

Plasma membrane (PM) H^+^-ATPase is a P-type H^+^-ATPase found in plants and fungi (Serrano, 1989). It hydrolyses ATP and pumps protons from the cytoplasm to the apoplast to establish electrochemical gradients across the plasma membrane, which are used to drive secondary transport processes including ion, lipid, sugar, and nutrient transport. PM H^+^-ATPase functions in many cellular and developmental processes in plants such as cell expansion, turgor regulation, cellular pH changes, tip growth, stomatal movement, and stress responses (Michelet and Boutry, 1995; Bibikova *et al.*, 1998; Palmgren, 1998, 2001; Morsomme and Boutry, 2000; Duby and Boutry, 2009; Lang *et al.*, 2014; Yan *et al.*, 2015).

PM H^+^-ATPase activity is tightly regulated during plant growth and development and in response to environmental changes. It is regulated by a number of endogenous and exogenous factors, such as hormones (auxin and abscisic acid), calcium, blue light, fungal elicitors, and stress stimuli (Johansson *et al.*, 1993; Niu *et al.*, 1993; Frías *et al.*, 1996; Kinoshita and Shimazaki, 1999; Janicka-Russak and Kłobus, 2007; Hayashi *et al.*, 2014; Spartz *et al.*, 2014). The C-terminal region of PM H^+^-ATPase is an auto-inhibitory domain, and PM H^+^-ATPase is constitutively activated when this region is truncated (Palmgren *et al.*, 1991). Phosphorylation of several Thr and Ser residues in the C-terminal region is crucial for the regulation of PM H^+^-ATPase activity (Rudashevskaya *et al.*, 2012; Haruta *et al.*, 2015). 14-3-3 proteins interact directly with the C-terminal region of the Thr-947 phosphorylation site to activate PM H^+^-ATPase (Jahn *et al.*, 1997; Svennelid *et al.*, 1999). Two phosphorylated residues in this region, Ser-931 and Ser-899, negatively regulate the activity (Duby *et al.*, 2009; Haruta *et al.*, 2014). The Arabidopsis Ser/Thr protein kinase PKS5 negatively regulates PM H^+^-ATPase activity by phosphorylating its Ser-931 residue, preventing its interaction with the 14-3-3 protein (Fuglsang *et al.*, 2007; Yang *et al.*, 2010). Another phosphorylation site, Ser-899, mediates RALF-FERONIA signal transduction (Haruta *et al.*, 2014).

Lipids play important roles in the regulation of PM H^+^-ATPase activity. *In vitro* studies suggest that PM H^+^-ATPase activity is affected by exogenous-added lipids in isolated vesicles, including fatty acids (FAs), phosphatidylserine (PS), phosphatidylinositol (PI), phosphatidylcholine (PC), phosphatidylglycerol (PG) and phosphatidic acid (PA) (Dufour and Goffeau, 1980; Palmgren *et al.*, 1988; Kasamo, 1990; Sánchez-Nieto *et al.*, 2011). Lysophospholipids stimulate PM H^+^-ATPase activity (Palmgren *et al.*, 1988; Palmgren and Sommarin, 1989; Kasamo, 1990; Pedchenko *et al.*, 1990; Gomès *et al.*, 1996; Wielandt *et al.*, 2015), and the activation depends on autoinhibitory N- and C-terminal domains of PM H^+^-ATPase (Wielandt *et al.*, 2015). These studies suggest that lipids are part of the regulatory machinery governing plant PM H^+^-ATPase activity. However, it is still unclear whether and how these lipids regulate activity in plants.

The levels of thousands of metabolites dynamically fluctuate throughout plant development and in response to various environmental stimuli. Some of these metabolites function as small, bioactive molecules that regulate plant growth and development, such as phytohormones (Lu and Xu, 2015). Many studies have been conducted on the pharmacological effects of plant-derived metabolites on human health; however, less is known about how these molecules function in plants and which targets they work on. In this study, based on the fact that salt stress rapidly activates PM H^+^-ATPase, we used a bio-guided isolation strategy to identify the bioactive endogenous compounds that activate PM H^+^-ATPase activity in Arabidopsis under salt stress. We isolated the free, unsaturated fatty acids C18:1, C18:2, and C18:3 from NaCl-treated cell fractions, evaluated them by spectrometric parameters, and found that they directly bind to and activate PM H^+^-ATPase.

## Materials and methods

### Plant materials

*Arabidopsis thaliana* Columbia (Col-0) wild-type, Nössen (NӦ) wild-type, *ssi2* mutant, and complemented lines *ProSSI2::SSI2-1* and *ProSSI2::SSI2-2* were used in this study. The NӦ and *ssi2* have been described previously (Kachroo *et al.*, 2001). The *ProSSI2::SSI2-1* and *ProSSI2::SSI2-2* were generated as follows: the 4421 bp *SSI2* genomic sequence (including 1262 bp upstream of the ATG start codon and 770 bp downstream of the TAA stop codon) was amplified from NӦ genomic DNA using primers 5′-GCGC*GAATTC*CTACATA TTTCTTCTGTGTGCTAAC-3′ containing an *Eco*RI restriction site and 5′-GC*GTCGAC*TCAACTTTGCTCTCACAATGATTC-3′ containing a *Sal*I restriction site. Then the *ProSSI2::SSI2* was cloned into the *Eco*RI and *Sal*I sites in the pCAMBIA1300 binary vector and the resulting construct was transformed into the *ssi2*.

### Extraction of endogenous small molecules

Arabidopsis Col-0 seeds were sterilized and sown in liquid 1/2 Murashige and Skoog (MS) medium with 10 g l^–1^ sucrose, grown under dark conditions and maintained shaking in a shaking incubator for 20 d, and treated with 150 mM NaCl or 1/2 MS for 6 h. The seedlings were gently squeezed to remove extra water and ground under liquid nitrogen. The material (1040 g treated with NaCl; 1170 g without salt treatment) was extracted in 5 l of methanol for 30 min under agitation, respectively. After filtration through a Buchner funnel, the material was re-extracted in 5 l of methanol. The two liquid extracts were combined and concentrated under reduced pressure using a rotary evaporator, resulting in 2.07 g of total crude extract (with salt treatment) and 2.20 g of total crude extract (without salt treatment).

### Isolation of plasma membrane vesicles and PM H^+^-ATPase activity assays

Plasma membrane-enriched vesicles were obtained from Arabidopsis seedlings with or without salt treatment using aqueous two-phase (Dextron-PEG3350) partitioning as described previously (Qiu *et al.*, 2002). Arabidopsis Col-0 seeds were sterilized, sown on solid MS medium with 25 g l^–1^ sucrose, and grown under continuous white light (light intensity of 50 μmol m^−2^ s^−1^) at 23 °C for 7 d. The seedlings were then transferred to soil for 4 weeks under a 16 h light (22 °C)–8 h dark (20 °C) cycle. For the salt treatment, NӦ, *ssi2*, *ProSSI2::SSI2-1*, and *ProSSI2::SSI2-2* seedlings were treated with 250 mM NaCl for 3 d prior to being collected for plasma membrane vesicles isolation. The isolation of plasma membrane vesicles was performed at 4 °C or on ice. Plants were homogenized in buffer (2 ml buffer per gram plant tissue) containing 10% (w/v) glycerol, 0.33 M sucrose, 0.2% (w/v) BSA, 5 mM dithiothreitol (DTT), 5 mM EDTA, 0.2% (w/v) casein, 5 mM ascorbate, 1 mM phenylmethylsulfonyl fluoride (PMSF), 0.6% (w/v) polyvinylpyrrolidone, 1× protease inhibitor, and 50 mM HEPES–KOH, pH 7.5. The homogenate was filtered through two layers of Miracloth and centrifuged at 12 000 *g* for 10 min. The supernatant was centrifuged for 1 h at 100 000 *g* to obtain a microsomal pellet, which was resuspended in a buffer containing 3 mM KCl, 0.33 M sucrose, 1 mM DTT, 1 mM PMSF, 0.1 mM EDTA, 1× protease inhibitor, and 5 mM K_2_HPO_4_–KH_2_PO_4_, pH 7.8. The suspension was added to a two-phase mixture to obtain a phase consisting of 6.2% (w/w) dextran T-500, and 6.2% (w/w) polyethylene glycol 3350 in 5 mM K_2_HPO_4_–KH_2_PO_4_ buffer, pH 7.8, containing 3 mM KCl and 0.33 M sucrose. The final upper phases were collected, diluted with resuspension buffer containing 10% (w/v) glycerol, 0.33 M sucrose, 0.1% (w/v) BSA, 0.1 mM EDTA, 1× protease inhibitor, 2 mM DTT and 20 mM HEPES–KOH, pH 7.5, and centrifuged for 1 h at 100 000 *g*. The pellet was collected and resuspended in the above-mentioned resuspension buffer plus 1 mM EDTA. Inside-out vesicles were produced by adding 0.05% (w/v) Brij58 to the medium as described previously (Johansson *et al.*, 1995). PM H^+^-ATPase activity was determined as described previously (Qiu *et al.*, 2002). H^+^-ATPase activity was evaluated based on the quenching (decrease) in fluorescence of quinacrine (a pH-sensitive fluorescent probe). The assays contained 5 mM quinacrine, 100 mM KCl, 3 mM MgSO_4_, 250 mM mannitol, 25 mM 1,3-bis[tris(hydroxylmethyl)methylamino]propane–HEPES, pH 6.5, and 50 μg ml^–1^ of vesicle protein. The reactions were mixed several times by inversion, incubated at 25 °C, and placed in a dark chamber in a fluorescence spectrophotometer (Hitachi F-7000). The assay was initiated by the addition of ATP (final concentration of 3 mM), and H^+^-ATPase activity was evaluated at excitation and emission wavelengths of 430 and 500 nm, respectively. At the end of the reaction, the protonophore carbonyl cyanide *m*-chlorophenyl hydrazone (CCCP; final concentration of 10 mM) was added to the assay solution to dissipate any remaining pH gradient.

PM H^+^-ATPase hydrolytic activity was determined by measuring the release of P_i_ from ATP (Sandstrom *et al.*, 1987). The resuspended plasma membrane vesicles (1 μg of protein) were added to 490 μl of assay buffer containing 30 mM MES/Tris (pH 6.5), 125 mM sucrose, 50 mM KCl, 5 mm MgSO_4_, 5 mM Na_2_ATP (pH 6.5), 0.1 mM (NH_4_)_6_Mo_7_O_24_, 50 mM KNO_3_, 1 mM NaN_3_, and 0.0125% (w/v) Triton X-100. The reaction proceeded for 20 min at 37 °C and was stopped by the addition of 1 ml of a stopping solution containing 5% (w/v) sodium dodecyl sulfate (SDS), 2% (w/v) H_2_SO_4_, and 0.5% (w/v) (NH_4_)_6_Mo_7_O_24_. Total P_i_ from ATP hydrolysis was measured spectrophotometrically by adding 50 μl of 10% (w/v) ascorbic acid and determining the absorbance at 660 nm after incubating at 45 °C for 20 min. Hydrolytic activity was evaluated in terms of μM P_i_ liberated per mg protein per min.

### H^+^ flux measurement

Net H^+^ fluxes were measured in the meristematic zone (approximately 120 μm from the root tip) of Arabidopsis roots using non-invasive micro-test technology (NMT; NMT100 Series, Younger USA LLC, Amherst, MA, USA; Xuyue (Beijing) Sci & Tech Co., Ltd, Beijing, China). The H^+^ concentration was evaluated by moving the H^+^-selective microelectrode between two positions close to the root at a programmable frequency in the range of 0.3–0.5 Hz. The micropipettes were front-filled with 15 μm columns of H^+^-selective liquid exchange cocktails (LIXs) (Fluka 95293). An Ag/AgCl wire electrode holder was inserted into the back of the electrode to make contact with the electrolyte buffer. The reference electrode was an Ag/AgCl half-cell connected to the assay solution. H^+^ fluxes were calculated by MageFlux, which is based on Fick’s law of diffusion. The samples were prepared and treated as described previously with some modifications (Fuglsang *et al.*, 2007). Arabidopsis NӦ, *ssi2*, *ProSSI2::SSI2-1*, and *ProSSI2::SSI2-2* seeds were sterilized and sown in solid MS medium plus 25 g l^–1^ sucrose and grown under continuous white light (light intensity of 50 μmol m^−2^ s^−1^) at 23 °C for 5 d. To observe the effect of exogenous addition of C18:1, C18:2, or C18:3 on the activation of PM H^+^-ATPase activity, the NӦ, *ssi2*, *ProSSI2::SSI2-1*, and *ProSSI2::SSI2-2* seedlings were pre-incubated in a buffer containing 0.5 mM KCl, 0.1 mM CaCl_2_, and 0.3 mM MES, pH 6.0, with the addition of 100 μM of C18:0, C18:1, C18:2, C18:3, or 0.1% dimethyl sulfoxide (DMSO) (v/v) for 20 min and assayed.

### Bio-guided isolation

The total methanol extract of seedlings with salt treatment and extract without salt treatment were separated simultaneously by normal-phase column chromatography on silica gel using a gradient elution of methanol–dichloromethane (DCM). The crude extract was loaded onto a silica gel column (200 ml) and fractioned by gradient elution with DCM as the starting elution solvent, followed by an increasing gradient of the polar solvent methanol from 5% to 100%. Every gradient elution needs 5× column volume of solvent. All subfractions were tested for their effect on PM H^+^-ATPase activity. The fractions that affected PM H^+^-ATPase activity were combined based on their thin layer chromatography (TLC) characteristics and further fractioned by repeated column chromatography, as shown in Supplementary Figs S1 (positive effect) and S2 (negative effect). Finally, the combined positive fraction in seedlings with salt treatment was fractioned by preparative high performance liquid chromatography–ultraviolet mass spectrometry (prep-HPLC-UV-MS); low resolution electrospray ionization mass spectrometry (LRESIMS) information about the compounds in active fractions is shown in Supplementary Fig. S3A–C.

### Structural elucidation of active compounds

Structural elucidation of the compounds in positive fractions was carried out using ^1^H-NMR (Varian, 400 MHz) and high resolution electrospray ionization mass spectrometry (HRESIMS; Thermo Fisher, Q-Exactive). Further verification was carried out via comparison with standards after derivation using gas chromatography–mass spectrometry (GC-MS) (see the method below).

### LC-MS analysis and preparation

The samples were analysed by HPLC–mass spectrometry on a Waters Auto Purification LC/MS system (3100 mass detector, 2545 binary gradient module, 2767 sample manager, 2998 photodiode array (PDA) detector, and system fluidics organizer). The system was equipped with a Waters C18 5 μm Xbridge analytical column (4.6mm×150 mm) or a preparative column (19mm×150 mm). The mobile phase consisted of acetonitrile (phase A) and H_2_O (phase B) at a flow rate of 1 ml min^–1^ in analytical mode and 15 ml min^–1^ in preparative mode. The preparative HPLC gradient elution conditions were as follows: 0 min, 50% A:50% B; 55 min, 73% A:27% B; 65 min, 82% A:18% B; and 75 min, 100% A:0% B. The fractions were collected in 15 ml glass tubes, with 10 ml elution sample in each glass tube, and column-wash elution solvent (100% A) was collected in a 500 ml round-bottomed flask. All fractions were solvent-evaporated, redissolved in methanol, and a PM H^+^-ATPase activity test performed.

### Fatty acid analysis

Direct transesterification of total fatty acids was performed as described previously (Miquel and Browse, 1992). Briefly, seedlings were ground under liquid nitrogen and lyophilized. Each lyophilized sample (30 mg) was combined with 3 ml of 3% H_2_SO_4_ in methanol in a screw-capped glass tube and incubated at 75 °C for 2.5 h. When the reaction was completed, 1 ml of 0.9% NaCl and 3 ml hexane were added, and the sample was mixed well. After standing for 1 h, the upper hexane layer was transferred to a new tube for analysis. GC-MS analysis of the methylated fatty acids was performed using an Agilent 7890 GC with a DB-23 column and 5975 mass selective detector. The oven conditions were as follows: 45 °C for 3 min, followed by 15 °C min^–1^ to 140 °C for 5 min, 1.5 °C min^–1^ to 144 °C for 12 min, 1.5 °C min^–1^ to 164 °C for 6 min, 1.5 °C min^–1^ to 170 °C for 0 min, and 20 °C min^–1^ to 230 °C for 5 min.

### Lipid-protein overlay assay

The fatty acids C18:0, C18:1, C18:2, and C18:3 were dissolved in DCM:methanol 1:1, and fatty acid test strips were prepared by spotting the indicated amounts of fatty acids onto a polyvinylidene difluoride membrane and allowing them to dry for 1 h at room temperature. The strips were incubated at room temperature for 2 h with 1 μg ml^–1^ of His fusion proteins in 5% skim milk–20 mM Tris–HCl (pH 8.0) plus 150 mM NaCl for blocking. After four washes, the bound proteins were detected using anti-His antibodies as previously described (Sun *et al.*, 2013). Bromophenol blue reagent was prepared by dissolving 0.04 g bromophenol blue in 100 ml ethanol.

### Fatty acid agarose affinity chromatography

Fatty acid sodium salts (C18:0, C18:1, C18:2, or C18:3, from J&K Scientific Ltd) were coupled to the Sepharose matrix (EAH Sepharose from GE Healthcare) as described previously (Peters *et al.*, 1973; Kim *et al.*, 2005; Mandal *et al.*, 2012). Briefly, Na-C18:1, Na-C18:2, and Na-C18:3 were dissolved in ddH_2_O, and Na-C18:0 was dissolved in 25% ethanol–ddH_2_O at a concentration of 100 μM. EAH-Sepharose, 1.5 volumes of 100 μM fatty acid sodium salts, and 1-ethyl-3-(3-dimethylaminopropyl)-carbodiimide (50 mg ml^–1^ of Sepharose) were mixed by inversion and incubated at 37 °C for 3 d. The Sepharose matrix was washed with 50% ethanol, followed by ethanol–75 mM NaH_2_PO_4_ (1:1), ethanol–50 mM NaOH (1:1), and phosphate buffered saline (PBS) at pH 7.0. The binding assay was performed as described previously (Kim *et al.*, 2005). Briefly, 2 μM His fusion protein in 200 μl PBS was incubated with 200 μl fatty acid–Sepharose at 37 °C for 1 h. The Sepharose was then washed with PBS and eluted with 50% ethanol. Binding proteins were analysed by immunoblot using anti-His antibodies.

### Membrane conductivity detection

The membrane conductivity assay was performed as previously described (Eich *et al.*, 2000). Briefly, ten Arabidopsis leaves (5-week-old plants) were pre-treated with 100 μM C18:0, C18:1, C18:2, or C18:3 or 150 mM NaCl for 12 h. A hole-puncher was used to obtain leaf tissue samples of the same size for each treatment. The leaf discs were immersed in ddH_2_O under vacuum for 10 min until the leaf discs were submerged, and electrolyte loss was measured after 3 h. The total electrolyte content of the leaves was determined by boiling the leaves for 10 min at 95 °C. Relative conductivity is the percentage of electrolyte loss *versus* total electrolyte content.

### Membrane fluidity detection

Five-day-old seedlings were treated with 100 μM C18:0, C18:1, C18:2, or C18:3 or 500 mM NaCl for 10 min, washed three times with ddH_2_O, and stained with 4 μM *N*-(3-triethylammoniumpropyl)-4-(6-[4-(diethylamino)phenyl]-hexatrienyl) pyridinium dibromide (FM 4-64, from Molecular Probes) for 10 min. The fluorescence recovery after photobleaching (FRAP) assay was conducted under a microscope, using Andor IQ software according to the manufacturer’s instructions. FRAP data analysis was performed as described previously (Goral *et al.*, 2010). Briefly, a plasma membrane region was selected and the fluorescence intensity of the region was measured in pre- and post-bleach images. The fluorescence intensity was analysed using ImageJ software. The fluorescence recovery rate is the percentage of post-bleach fluorescence intensity versus pre-bleach fluorescence intensity.

### Antibody and protein-blot analyses

The AHA2 centerloop and C-terminus coding sequences were amplified by RT-PCR using primer pairs 5′-CG*GGATCC*GCAGG AATGGATGTCCTGTGC-3′ and 5′-CG*GAATTC*TTAGA GCACGATATCTGAAGCACC-3′, and 5′-CG*GGATCC*GCGT GGCTCAACTTGTTTGAGAAC-3′ and 5′-CG*GAATTC*CTACA CAGTGTAGTGACTG-3′, respectively. The PCR products were cloned into the pET-28a-SMT3 vector to generate His-SMT3-AHA2 centerloop or His-SMT3-AHA2 C-terminus fusion protein. All plasmids were verified by sequencing. The recombinant proteins were expressed purified in *E. coli* bacterial cells as described in the manufacturer’s instructions.

Polyclonal antibody was raised against the AHA2 centerloop region (321–620 amino acid residue) from an antiserum of mouse. The AHA2 centerloop recombination protein can be recognized by the anti-AHA2 antibody; as a negative control the AHA2 C-terminus recombination protein cannot be recognized by the antibody (Supplementary Fig. S4A). The anti-H^+^-ATPase AHA2 antibody recognized not only AHA2 but also other plasma membrane H^+^-ATPase isoforms in Arabidopsis (Supplementary Fig. S4B). Protein samples (plasma membrane vesicles) were mixed with 5× SDS loading buffer (250 mM Tris–HCl pH 6.8, 30% glycerol, 10% SDS, 0.01% bromophenol blue and 200 mM dithiothreitol) followed by boiling for 5 min. Samples were loaded onto 12% SDS-PAGE gels and subjected to electrophoresis in denaturing SDS running buffer (3.03 g l^−1^ Tris, 1.0 g l^−1^ SDS, 14.4 g l^−1^ glycine). Proteins were transferred to nitrocellulose membranes. Membranes were probed with anti-AHA2 antisera at a 1:1000 dilution. Secondary antibody was horseradish peroxidase-conjugated goat anti-mouse IgG, and diluted at a 1:5000.

## Results

### Identification of fractions that activate PM H^+^-ATPase activity using a bio-guided isolation protocol

To investigate the endogenous small molecules involved in activating/deactivating PM H^+^-ATPase activity, we first utilized a bio-guided isolation procedure to isolate bioactive compounds. PM H^+^-ATPase has relatively low activity under normal growth conditions; however, this activity increases significantly in response to salt stress (Yang *et al.*, 2010). To determine whether small molecules are involved in this increased activation, we used our newly developed bio-guided isolation technique to fractionate crude extracts of Arabidopsis Col-0 seedlings with or without NaCl treatment. In total, 25 fractions were collected (based on polarity; Supplementary Figs S1 and S2), solvent-evaporated, and dissolved in methanol.

**Fig. 1.**
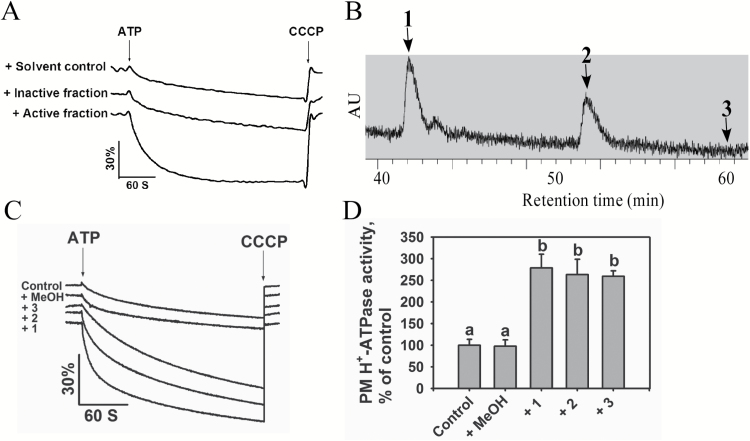
Identification of fractions that induce PM H^+^-ATPase activity using a bio-guided isolation protocol. (A) PM H^+^-ATPase activity measured in the vesicles in the presence of fractions. Plasma membrane vesicles were obtained from 5-week-old Arabidopsis (ecotype Col-0) seedlings. The reaction (proton-pumping resulting in intravesicular acidification and a pH gradient) was initiated by the addition of 3 mM ATP, and the pH gradient collapsed from the addition of 10 μM carbonyl cyanide *m*-chlorophenyl hydrazone (CCCP). (B) HPLC-UV profiling of active fractions 1, 2, and 3 in the process of preparation. (C) PM H^+^-ATPase activities measured in vesicles in the presence of fractions 1, 2, and 3. (D) Comparison of PM H^+^-ATPase activities in (C). Fraction-dependent change values in proton-pumping activity were calculated by setting the activity of the control to 100%. The data in (D) represent means±standard deviation (SD) of five replicates. Student’s *t*-test was used to analyse the statistical significance; significant differences (*P*≤0.05) in (D) are indicated by different lower-case letters.

To perform the PM H^+^-ATPase activity assay, we prepared plasma membrane vesicles from 5-week-old soil-grown Col-0 plants without NaCl treatment (Yang *et al.*, 2010). We evaluated the purity and H^+^-transport competency of the isolated vesicles and found them to be transport-competent (Supplementary Table S1 and Supplementary Fig. S5). We then pre-incubated the plasma membrane vesicles with each small-molecule fraction at a concentration of 10 μg ml^–1^ and evaluated the effect of each fraction on PM H^+^-ATPase activity (Fig. 1A). The fractions A2 and A3 isolated from the seedlings with salt treatment enhanced PM H^+^-ATPase activity, whereas the A2 and A3 fractions from the seedlings without salt treatment had no such effect (Supplementary Figs S1 and S2). Active fractions A2 and A3 were combined and further separated into four new fractions, B1–B4, using Sephadex LH-20 column chromatography, in which, fraction B2 was shown to activate PM H^+^-ATPase activity. We also separated the A2 and A3 fractions from the seedlings without salt treatment into four new fractions and none of them had an effect on the activity (Supplementary Fig. S2). We then separated the active fraction B2 from seedlings with salt treatment into five fractions by gel filtration on silica gel eluted with PE (petroleum ether)–acetone (10:1), in which fractions C2, C3, and C4 were shown to activate PM H^+^-ATPase activity. We then combined fractions C2, C3, and C4 and subjected them to further separation using Sephadex LH-20 column chromatography eluted with methanol. The resulting subfractions, D2 and D3, increased PM H^+^-ATPase activity. We separated fractions D2 and D3 by normal-phase column chromatography on silica gel using PE–DCM (1:1) as the elution solvent and found that the resulting fraction, E8, activated PM H^+^-ATPase activity (Supplementary Fig. S1). Finally, we separated fraction E8 by prep-HPLC-UV-MS and identified three active fractions, 1, 2, and 3 (Fig. 1B), all of which increased PM H^+^-ATPase activity (Fig. 1C, D). Active compounds in fractions 1 and 2 were further elucidated by ^1^H-NMR spectra, HRESIMS and GC-MS. An active compound in fraction 3 had a weak signal in ^1^H-NMR, and was further elucidated by HRESIMS and GC-MS.

### Structural elucidation of compounds in active fractions 1, 2 and 3

**Fig. 2.**
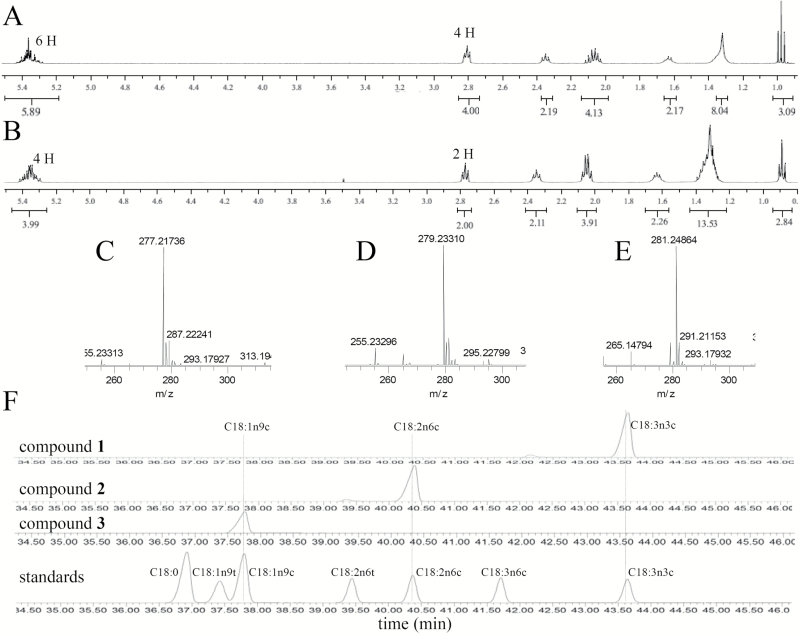
Structural elucidation of compounds in fractions 1, 2, and 3. (A) ^1^H-NMR spectrum of compound in fraction 1 (CDCl_3_, 400 MHz). (B) ^1^H-NMR spectrum of compound in fraction 2 (CDCl_3_, 400 MHz). (C) HRESIMS of compound in fraction 1 (ESI^−^). (D) HRESIMS of compound in fraction 2 (ESI^−^). (E) HRESIMS of compound in fraction 3 (ESI^−^). (F) Comparison of methyl derivatives of compounds in fractions 1, 2, and 3 with a standard fatty acid methyl ester mix.

LRESIMS (electrospray ionization in negative ion mode; ESI^−^) revealed values of *m*/*z* of 277 for a compound in fraction 1, 279 for a compound in fraction 2, and 281 for compound in fraction 3, suggesting that these molecules have similar structures (Supplementary Fig. S3A–C). The molecular formula of the compound in fraction 1 was determined to be C_18_H_29_O_2_ by HRESIMS at *m*/*z* 277.21736 [M–H]^−^ (calculated 277.21676, Fig. 2C), which indicates three degrees of unsaturation. The ^1^H-NMR spectra of the active compound in fraction 1 (Fig. 2A and Supplementary Table S2) displayed six hydrogens (Hs) at δ 5.36 ppm, which correlated with three degrees of unsaturation based on its HRESIMS data. Four protons at δ 2.05 ppm indicated the presence of two α-H groups next to one double bond, and four protons at δ 2.81 ppm indicated the presence of two α-H groups between two double bonds. A carbonyl moiety was present, with two α-H protons at δ 2.35 ppm and two β-H protons at δ 1.65 ppm next to the carbonyl moiety. This information, combined with the finding that methyl Hs at δ 0.98 ppm and high-abundance alkyl Hs at δ 1.31 ppm were observed in the ^1^H-NMR spectrum, indicated that the compound in active fraction 1 might be C18:3. GC-MS analysis of the methyl derivative of the compound in fraction 1 showed the same retention time at 43.6 min (Fig. 2F) and the same characteristic mass fragment ions (data not shown) as the commercially available methyl-linolenate standard. On this basis, the compound in active fraction 1 was identified as C18:3 (C18:3n3c).

The molecular formula of the compound in fraction 2 was determined to be C_18_H_31_O_2_ by HRESIMS at *m*/*z* 279.23310 [M–H]^−^ (calculated 279.23241, Fig. 2D), indicating two degrees of unsaturation. The ^1^H-NMR data for the compound in fraction 2 (Fig. 2B and Supplementary Table S2) were similar to those of the compound in fraction 1 except for the presence of four double-bond Hs at δ 5.36 ppm and two α-H protons between the two double bonds at δ 2.81 ppm, indicating that two double bonds exist in the structure. Based on structural analysis of the compound in fraction 2, we postulated that it might be C18:2. To further verify the structure of the compound in fraction 2, we compared its methylated derivative with standard methyl-C18:2 via GC-MS. Both showed the same characteristic mass fragment ions and the same retention time at 40.35 min (Fig. 2F). Thus, the compound in active fraction 2 was identified as C18:2 (C18:2n6c).

The molecular formula for the compound in fraction 3 was determined to be C_18_H_33_O_2_ based on the HRESIMS peak at *m*/*z* 281.24864 [M–H]^−^ (calculated 281.24806, Fig. 2E), with two more Hs than the compound in fraction 2, suggesting that compound 3 might be C18:1. We compared its methylated derivative with standard methyl-C18:1, both of which showed the same retention time at 37.8 min (Fig. 2F). Thus, we confirmed that the compound in active fraction 3 is C18:1 (C18:1n9c).

### The effect of exogenous C18:1, C18:2, or C18:3 addition on PM H^+^-ATPase activity

**Fig. 3.**
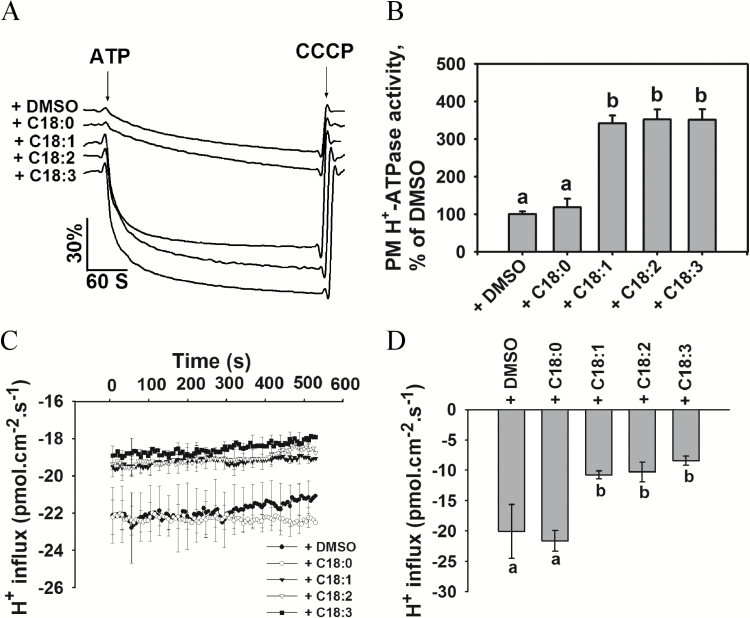
Exogenous addition of fatty acids increases H^+^ extrusion. (A) PM H^+^-ATPase activities measured in vesicles in the presence of the compounds. Plasma membrane vesicles were isolated from 5-week-old Arabidopsis (ecotype Col-0) seedlings. (B) Comparison of PM H^+^-ATPase activities in (A). (C) Net H^+^ fluxes in root tips of Col-0 plants in the presence of the compounds. The non-invasive microsensing system technique was used to monitor ion flux. After the roots were incubated in buffer (0.5 mM KCl, 0.1 mM CaCl_2_, and 0.3 mM MES, pH 6.0) in the presence of a DMSO control or 100 μM of one of the compounds for 20 min, the transient net H^+^ fluxes were recorded in the buffer mentioned above. (D) Calculated net H^+^ fluxes from (C). The data in (B–D) represent means±SD of five replicates. The three biological replicates displayed similar results. Student’s *t*-test was used to analyse the statistical significance; significant differences (*P*≤0.05) in (B, D) are indicated by different lower-case letters.

To exclude the possibility that endogenous C18:1, C18:2, and C18:3 isolated from Arabidopsis may contain other substances involved in activating PM H^+^-ATPase activity, we performed a verification experiment using commercially available standard samples. We pre-incubated the PM vesicles with 100 μM C18:0, C18:1, C18:2, or C18:3, or 0.1% DMSO (v/v) and measured the PM H^+^-ATPase activity. As shown in Fig. 3A, B, exogenous addition of C18:1, C18:2, or C18:3 to the vesicles caused a significant increase in H^+^-transport activity compared with the solvent control (0.1% DMSO, v/v). However no obvious effect on PM H^+^-ATPase activity was observed in the presence of C18:0. When we evaluated the effect of unsaturated fatty acids on hydrolytic activity, the presence of C18:1, C18:2, or C18:3 in the assay solution resulted in an increase in hydrolytic activity, but incubation with C18:0 had no obvious effect on this activity (Supplementary Table S3).

We further verified the effects of unsaturated fatty acids on H^+^ fluxes in Arabidopsis roots using the non-invasive microsensing system (NMS), which can be used to monitor transport of various ions and molecules in intact samples (Xu *et al.*, 2006). Net H^+^ fluxes were measured in the root apex of seedlings pre-incubated with 100 μM C18:0, C18:1, C18:2, or C18:3, or 0.1% DMSO (v/v). The H^+^ influxes were reduced after treatment with C18:1, C18:2, or C18:3 (Fig. 3C, D), but C18:0 did not affect H^+^ fluxes in the root compared with the DMSO control. Taken together, these results suggest that C18:1, C18:2, and C18:3 exert a positive regulatory effect on PM H^+^-ATPase activity.

### C18:1, C18:2, and C18:3 bind to the C-terminus of AHA2

To understand how the unsaturated fatty acids regulate PM H^+^-ATPase activity and to identify which part of AHA2 is involved in this regulation, we performed binding assays between C18:1, C18:2, or C18:3 and AHA2 peptides. Specifically, DNA fragments encoding the centerloop (amino acids 321–620) and C-terminus (amino acids 849–948) of AHA2 were cloned into the pET-28a-SMT3 vector and the resulting plasmids were transformed into *E. coli* strain BL21. The recombinant proteins were then purified.

**Fig. 4.**
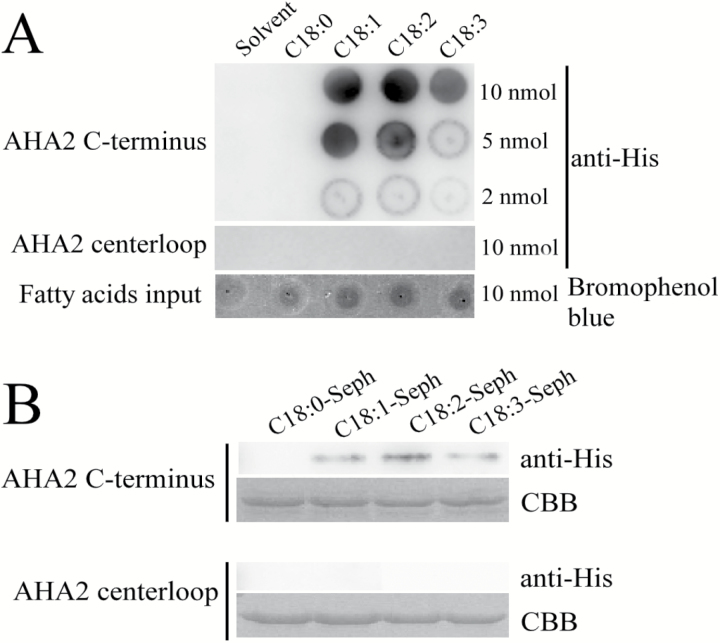
C18:1, C18:2, and C18:3 bind to the C-terminus of PM H^+^-ATPase AHA2. (A) Lipid–protein overlay experiment between AHA2 peptides (C-terminus and centerloop) as His fusion proteins extracted from *E. coli* and C18:1, C18:2, or C18:3. The solvent (DCM:methanol, 1:1) and C18:0 were used as controls. The amount that each lipid spot contained is shown on the right side. The upper lane shows the interaction between the AHA2 C-terminus and the fatty acids. The middle lane shows the interaction between the AHA2 centerloop and the fatty acids. The lower lane shows the fatty acid input control, which was stained by bromophenol blue. The experiment was repeated three times with similar results. (B) C18:0, C18:1, C18:2, and C18:3 agarose affinity chromatography experiment performed with the AHA2 peptides (C-terminus and centerloop) as His fusion proteins extracted from *E. coli*. The fatty acid–Sepharose matrixes were incubated with the peptides (C-terminus and centerloop) and washed with PBS, and the bound peptides were eluted and detected by immunoblot using anti-His antibodies. The upper lanes show detection using the C-terminus and the lower lanes show detection using the centerloop. The input control of each experiment was detected with Coomassie brilliant blue staining (CBB). The experiment was repeated three times with similar results.

We used a lipid–protein overlay experiment to investigate the interaction between C18:0, C18:1, C18:2, or C18:3 and the peptides. The unsaturated fatty acids C18:1, C18:2, and C18:3 physically interacted with the C-terminus of AHA2, but not its centerloop (Fig. 4A). By contrast, C18:0 did not interact with the centerloop or the C-terminus of AHA2.

To further verify the interaction between C18:1, C18:2, or C18:3 and the C-terminus of PM H^+^-ATPase, we prepared C18:0–, C18:1–, C18:2–, and C18:3–Sepharose columns, which we loaded with the peptides of the C-terminus or centerloop of AHA2. The C-terminus was pulled down by the C18:1, C18:2, and C18:3 Sepharose matrix, but the centerloop was not (Fig. 4B). Consistently, none of the AHA2 peptides were pulled down by the C18:0 matrix (Fig. 4B). These results suggest that C18:1, C18:2, and C18:3 activate PM H^+^-ATPase activity by directly interacting with its C-terminus.

### PM H^+^-ATPase activity is decreased in an Arabidopsis mutant with low levels of unsaturated fatty acids

**Fig. 5.**
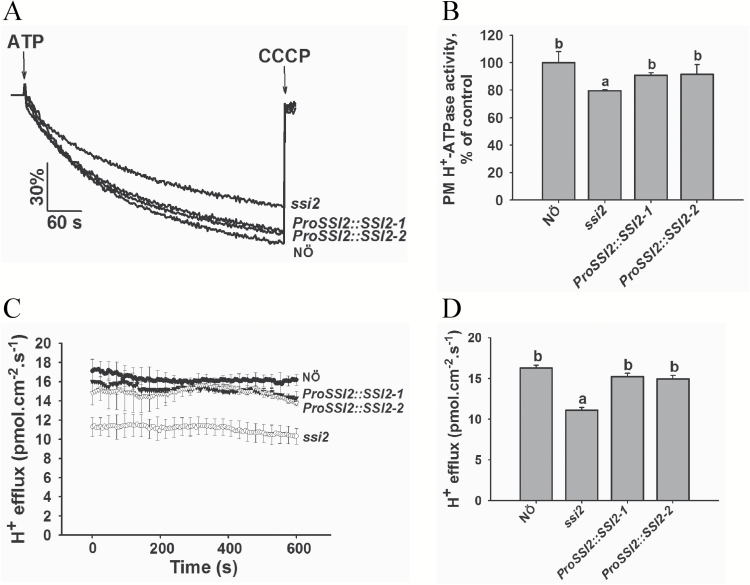
PM H^+^-ATPase activity is reduced in the *ssi2* mutant. (A) PM H^+^-ATPase activity was measured in the vesicles of NӦ, *ssi2*, *ProSSI2::SSI2-1*, and *ProSSI2::SSI2-2* seedlings. Plasma membrane vesicles were isolated from 5-week-old Arabidopsis (ecotype Col-0) seedlings treated with 250 mM NaCl for 3 d. (B) Comparison of PM H^+^-ATPase activity in (A). (C) The net H^+^ effluxes in root tips of NӦ, *ssi2*, *ProSSI2::SSI2-1*, and *ProSSI2::SSI2-2* seedlings. The non-invasive microsensing system technique was used to monitor ion flux. After the roots were incubated under alkaline conditions (0.5 mM KCl, 0.1 mM CaCl_2_, 0.3 mM MES, and 75 mM NaCl, pH 8.1) for 20 min, the transient net H^+^ fluxes were recorded in the same buffer. (D) Calculated net H^+^ effluxes from (C). The data in (B–D) represent means±SD of five replicates. The three biological replicates displayed similar results. Student’s *t*-test was used to analyse the statistical significance; significant differences (*P*≤0.05) in (B, D) are indicated by different lower-case letters.

In Arabidopsis, *SSI2* encodes a fatty acid desaturase involved in the desaturation of fatty acid C18:0 to C18:1. To determine the genetic linkage between unsaturated fatty acids and PM H^+^-ATPase activity, we obtained the *ssi2* mutant (in the NӦ background) (Kachroo *et al.*, 2001) and generated *ProSSI2*:*SSI2* transgenic rescue lines (*ProSSI2::SSI2-1* and *ProSSI2::SSI2-2*) in this mutant background. The levels of C18:1, C18:2, and C18:3 were significantly reduced in the *ssi2* mutant compared with wild-type, whereas the *ProSSI2::SSI2-1* and *ProSSI2::SSI2-2* transgenic plants had levels of unsaturated fatty acids similar to those of wild-type (Supplementary Fig. S6). We isolated plasma membrane vesicles from leaves of wild-type NӦ, *ssi2*, *ProSSI2::SSI2-1*, and *ProSSI2::SSI2-2* plants treated with 250 mM NaCl for 3 d and measured PM H^+^-ATPase H^+^-transport and hydrolytic activity. These activities were lower in vesicles isolated from the *ssi2* mutant than those of NӦ, whereas in the *ProSSI2::SSI2-1* and *ProSSI2::SSI2-2* genotypes, PM H^+^-ATPase H^+^-transport and hydrolytic activity were restored to wild-type levels (Fig. 5A, B and Supplementary Table S4). We monitored the protein level of PM H^+^-ATPase via immunoblot analysis using H^+^-ATPase AHA2 antibody and found no significant difference in AHA2 levels among NӦ, *ssi2*, *ProSSI2::SSI2-1*, and *ProSSI2::SSI2-2* plants (Supplementary Fig. S7).

We measured net H^+^ fluxes in the root apices of 7-day-old NӦ, *ssi2*, *ProSSI2::SSI2-1*, and *ProSSI2::SSI2-2* seedlings using the NMS method. Specifically, we pretreated the seedlings under salt–alkaline conditions (0.5 mM KCl, 0.1 mM CaCl_2_, 0.3 mM MES, 75 mM NaCl, pH 8.1) to activate PM H^+^-ATPase activity and evaluated the difference in net H^+^ efflux. The rate of transmembrane H^+^ efflux was significantly lower in the *ssi2* mutant than in wild-type NӦ, whereas no difference in net H^+^ efflux was detected among NӦ, *ProSSI2::SSI2-1*, and *ProSSI2::SSI2-2* plants (Fig. 5C, D). These results further support the notion that PM H^+^-ATPase activity is activated by unsaturated fatty acids C18:1, C18:2, and C18:3.

### Exogenous addition of C18:1, C18:2, or C18:3 does not influence membrane conductivity or fluidity

To exclude the possibility that C18:1, C18:2, and C18:3 influence membrane properties, we tested their effects on membrane conductivity and membrane fluidity. In our membrane conductivity assay, samples treated with 100 μM C18:0, C18:1, C18:2, or C18:3 showed similar relative conductivity values to that of the solvent control (0.1% DMSO, v/v); however, treatment with 150 mM NaCl led to a significantly larger change in relative conductivity compared with unsaturated fatty acid treatments (Supplementary Fig. S8A). In the membrane fluidity assay, we used fluorescence recovery after photobleaching (FRAP) to investigate the effect of C18:0, C18:1, C18:2, or C18:3 on membrane fluidity in Arabidopsis seedlings. Treatment with 100 μM C18:0, C18:1, C18:2, or C18:3 had similar effects on membrane fluidity to that of the solvent control (0.1% DMSO, v/v) (Supplementary Fig. S8B–D). However, NaCl treatment reduced membrane fluidity. This result is consistent with the previous finding that C18:2 does not influence membrane fluidity (Lin *et al.*, 2011).

## Discussion

Many metabolites are generated in plants during metabolism, and their levels are altered throughout development and in response to environmental stimuli. Metabolites function in many physiological processes and can also serve as cofactors with stimulatory or inhibitory effects on enzymes. In plants, the biological functions of many metabolites have not yet been discovered.

In this study, we fractionated small molecules from NaCl-treated or untreated Arabidopsis seedlings according to their polarity and molecular mass and used a PM H^+^-ATPase activity-guided assay to identify fractions that activate or deactivate PM H^+^-ATPase activity. Using this strategy, we obtained three small molecules, unsaturated fatty acids C18:1, C18:2, and C18:3. We performed a direct binding assay that showed that unsaturated fatty acids C18:1, C18:2, and C18:3 bound to the C-terminus of PM H^+^-ATPase and stimulated its activity. We also performed a PM H^+^-ATPase activity assay in a mutant with low levels of unsaturated fatty acids, as well as transgenic rescued lines, which gave the genetic evidence that the unsaturated fatty acids play a role in activating PM H^+^-ATPase activity. Salt stress triggers many plant responses, including osmotic, ion and ROS signals, and also causes many physiological changes, including membrane properties. It is believed that Na^+^ causes membrane destabilization by reducing the hydration of fatty acids and displacing the Ca^2+^ in the membranes, which in turn change membrane properties (Cramer *et al.*, 1985; Lynch *et al.*, 1987). However, the unsaturated fatty acids do not affect membrane properties, indicating that they function in plant salt response by a specific pathway. It is still unclear how these unsaturated fatty acids activate H^+^-ATPase. It is possible that the binding of the unsaturated fatty acids to the C-terminus changes its conformation and in turn alters the inhibition activity of PM H^+^-ATPase, or that the binding changes the phosphorylation status of H^+^-ATPase, repressing Ser-931 phosphorylation by PKS5 and increasing Thr-947 phosphorylation to activate PM H^+^-ATPase. Further studies are needed to investigate these possibilities.

Our bio-guided isolation result is in agreement with previous studies that exogenous addition of free fatty acids enhances PM H^+^-ATPase activity (Kasamo, 1990; Palmgren *et al.*, 1988), which supports the reliability of our bio-guided method. It is also reported that various lysophospholipids can also enhance PM H^+^-ATPase activity (Kasamo, 1990; Palmgren *et al.*, 1988). Lysophosphatidylcholine (LPC) has been studied in yeast for its possible function in regulating PM H^+^-ATPase activity through both the N- and C-terminus of PM H^+^-ATPase (Wielandt *et al.*, 2015). However, genetic and biochemical information is lacking on whether these small molecules directly bind to PM H^+^-ATPase and affect its activity in plants.

Phospholipids, one of the major classes of metabolites in plants, play critical roles in plant development and stress responses (Xue *et al.*, 2007). Using our bio-guided method, we also found the existence of phosphatidylcholine (PC) in active fractions (A22) from both of the salt-treated and untreated seedlings (Supplementary Fig. S9A–D). PC (particularly PC with unsaturated acyl chains) was reported to activate PM H^+^-ATPase (Kasamo, 1990), and the commercially standard PC sample can activate PM H^+^-ATPase (Supplementary Fig. S9E). The PC is enriched in both salt-treated and untreated seedlings and the free unsaturated fatty acids were enriched only in the samples of the salt-treated seedlings, but in different fractions from those that activate PM H^+^-ATPase activity. Based on our small-molecule fractionation method, the free fatty acids are unlikely to result from lipolysis during the fractionation process; however, they might be generated from some phospholipids via phospholipases A (PLAs) in plants.

There is a complex network of different phospholipid signaling pathways, and phospholipids exert their physiological function by dynamic conversion of different phospholipids. Although various lysophospholipids and free unsaturated fatty acids appear to have a similar ability to activate plant PM H^+^-ATPase activity, their specificity is yet to be determined. In plants, lysophospholipids and free unsaturated fatty acids are generated from phospholipids simultaneously, and either differences in binding affinity or local changes in small molecule concentrations may result in this binding specificity and guide plant responses. It is possible that different lipids play a specific role to fine tune PM H^+^-ATPase activity in a timing- and locality-dependent manner under different stress stimuli. Further studies to determine when and where lysophospholipids and free unsaturated fatty acids are produced may help us to understand the difference in the regulation.

Several studies have investigated total fatty acid contents in plants (Kodama *et al.*, 1995; Murakami *et al.*, 2000; Kachroo *et al.*, 2001; Devaiah *et al.*, 2007; Zhang *et al.*, 2009; Zhang *et al.*, 2012). In the current study, the content of total C18 unsaturated fatty acids did not change under salt treatment (Supplementary Fig. S10). The free unsaturated fatty acids were only identified in the fractions isolated from more than 1 kg of seedlings treated with NaCl, but not from the same amount of seedlings without NaCl treatment, suggesting that the free unsaturated fatty acid content is very low and induced by salt stress. We attempted to measure the content of free unsaturated fatty acids in Arabidopsis with 0.5 g of material using two reported methods: GC-MS analysis after conversion to Weinreb amides and direct high-performance thin-layer chromatography analysis, which are used in animal and yeast cells (Maeda *et al.*, 2013, 2014; Ubhayasekera *et al.*, 2013); however, we failed to quantify the content of free unsaturated fatty acids. Perhaps the content of free unsaturated fatty acids in small samples is too low to reach the quantification limit of these two methods.

PLAs play important roles in the generation of unsaturated free fatty acids. They consist of a superfamily of enzymes that catalyse the hydrolysis of the ester bonds of phospholipids to generate lysophospholipids and free fatty acids (Ryu, 2004). PLAs function in many physiological processes, including cell elongation, shoot gravitropism and stomatal opening (Lee *et al.*, 2003; Seo *et al.*, 2008), which require the stimulation of PM H^+^-ATPase activity. These results suggest that PLAs may also be involved in the regulation of PM H^+^-ATPase activity. The degree of unsaturation of free fatty acids depends on desaturase in plants. In our study, SSI2, a plastid-located desaturase (Wu *et al.*, 2009), is involved in the regulation of PM H^+^-ATPase activity. PLA_1_ (class I, II, and III), hydrolysing fatty acids at the sn-1 position, is predicted to localize in chloroplasts, cytosol, and mitochondria based on sequence analysis (Ishiguro *et al.*, 2001; Ryu, 2004; Wang *et al.*, 2012); sPLA_2_ in Arabidopsis (AtsPLA_2_α, AtsPLA_2_β, AtsPLA_2_γ, and AtsPLA_2_δ), hydrolysing fatty acids at the sn-2 position, is reported to localize in apoplasts, Golgi bodies and/or endoplasmic reticulum (Seo *et al.*, 2008; Froidure *et al.*, 2010; Kim *et al.*, 2011; Jung *et al.*, 2012). It is reported that PLAs in plant cells can be relocalized (Froidure *et al.*, 2010; Jung *et al.*, 2012). PLA_2_α is translocated from cytoplasmic vesicles to nucleus mediated by AtMYB30 (Froidure *et al.*, 2010). It can also be translocated from Golgi bodies to apoplasts during development and bacterial infection in Arabidopsis (Jung *et al.*, 2012). Phospholipids and free fatty acids could be translocated in the cell through membrane contact sites and vesicle traffic (Li *et al.*, 2016). Although many transport proteins are involved in the lipid translocation, no exact protein is reported to transport free fatty acids to plasma membrane (Li *et al.*, 2016). It is possible that either unknown proteins are involved in this process or relocated PLAs produce free fatty acids in plasma membrane to activate PM H^+^-ATPase.

The levels of LPC and lysophosphatidic acid (LPA) increased markedly after hyperosmotic shock in *Dunaliella salina* (Einspahr *et al.*, 1988), and LPA accumulated in a dose- and time-dependent manner after NaCl treatment (Meijer *et al.*, 2001) in *Chlamydomonas*. Because free unsaturated fatty acids are generated with LPA and LPC simultaneously, it is possible that free fatty acid levels are also elevated under osmotic and salt stress. The expression of *SSI2* was induced after salt treatment (Supplementary Fig. S11). These results suggest that the content of free unsaturated fatty acids increased under salt stress.

The expression of genes involved in lipid metabolism is regulated by various environmental stimuli. In Arabidopsis, salt stress induces the expression of genes encoding fatty acid desaturase-2 (*FAD2*), fatty acid desaturase-6 (*FAD6*), and phospholipase D, which hydrolyses phospholipids to produce phosphatidic acid (Zhang *et al.*, 2009, 2012; Yu *et al.*, 2010), and low temperature induces the expression of fatty acid desaturase-8 (*FAD8*) (Gibson *et al.*, 1994). In tomato, the expression of the fatty acid desaturase-7 gene (*LeFAD7*) is induced by chilling stress but reduced by high temperature (Liu *et al.*, 2006). These findings suggest that different lipid products may play different roles in the regulation of physiological function in facing multiple environments. The technique developed in this study can be used as a starting point to investigate the roles of these and other small molecules that activate PM H^+^-ATPase in more detail.

## Supplementary data

Supplementary data are available at *JXB* online.

Fig. S1. The bio-guided isolation procedure for seedlings with salt treatment.

Fig. S2. The bio-guided isolation procedure for seedlings without salt treatment.

Fig. S3. LRESIMS spectrum of compounds in fractions 1, 2, and 3.

Fig. S4. Availability of the AHA2 antibody.

Fig. S5. Proton transport competency of the isolated vesicles.

Fig. S6. Fatty acid analyses of NӦ, *ssi2*, *ProSSI2::SSI2-1*, and *ProSSI2::SSI2-2* seedlings.

Fig. S7. PM H^+^-ATPase AHA2 protein levels in NӦ, *ssi2*, *ProSSI2::SSI2-1*, and *ProSSI2::SSI2-2* seedlings.

Fig. S8. Exogenous application of C18:1, C18:2, or C18:3 does not influence membrane conductivity and membrane fluidity.

Fig. S9. The fraction containing PC activates PM H^+^-ATPase activity.

Fig. S10. Total fatty acid analyses of col-0 under salt stress.

Fig. S11. Expression of *SSI2* after salt treatment.

Table S1. Plasma membrane purity determination.

Table S2. ^1^H NMR spectral data for compounds in fractions 1 and 2 (CDCl_3_, 400 MHz).

Table S3. Effect of fatty acids on plasma membrane H^+^-ATPase hydrolytic activity.

Table S4. Plasma membrane H^+^-ATPase hydrolytic activity of NӦ, *ssi2*, *ProSSI2::SSI2-1*, and *ProSSI2::SSI2-2* seedlings.

## Supplementary Material

supplementary_figures_S1_S11_tables_S1_S4
