# Supplementary material for: A bioassay-guided fractionation system to identify endogenous small molecules that activate plasma membrane H+-ATPase activity in Arabidopsis
Source: J Exp Bot. 2017 Jun 3;68(11):2951–62. doi: 10.1093/jxb/erx156 (PMC5853834; doi:10.1093/jxb/erx156)
Supplement: supplementary_figures_S1_S11_tables_S1_S4 [file erx156_suppl_supplementary_figures_s1_s11_tables_s1_s4.pdf]

Supplemental Figure 1

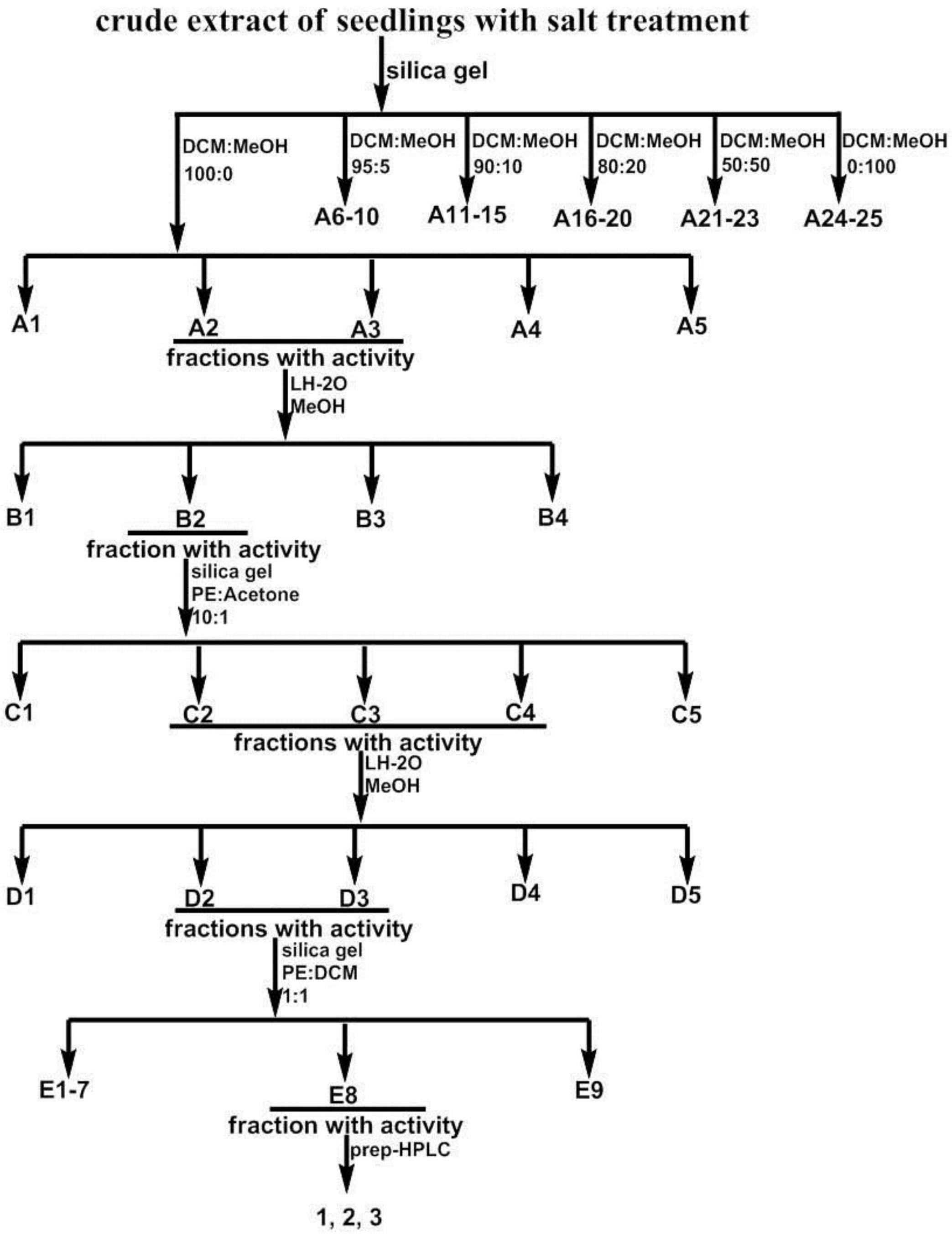

Supplemental Figure 1. The bio-guided isolation procedure of the seedlings with salt treatment.

The MeOH extract (2.07 g) was subjected to a silica gel normal-phase column, eluting with a stepwise gradient solvent of DCM-MeOH (100:0, 95:5, 90:10, 80:20, 50:50, 0:100) to give 25 fractions (FrA1-FrA25). All fractions were applied on PM H^+-ATPase activity test. FrA2-FrA3 with activity were combined and subjected to a Sephadex LH-20 column eluting with MeOH to give four fractions (FrB1-FrB4). All these four fractions were applied on PM H^+-ATPase activity test and fraction B2 showed activity in stimulating PM H^+-ATPase activity. Fraction B2 was separated on normal-phase column chromatography eluting with a isocratic gradient solvent of PE:Acetone 10:1 to give five fractions (FrC1-FrC5). All these five fractions were applied on PM H^+-ATPase activity test. Fractions C2 to fraction C4 with activity were combined and subjected to a Sephadex

LH-20 column eluting with MeOH to give five fractions (FrD1-FrD5). All these five fractions were applied on PM H^+-ATPase activity test. Fractions D2 and fraction D3 with activity were combined and separated on normal-phase column chromatography eluting with a isocratic gradient solvent of PE:DCM 1:1 to give nine fractions (FrE1-FrE9). All these nine fractions were applied on PM H^+-ATPase activity test and fraction E8 showed activity in stimulating PM H^+-ATPase activity. Fractions E8 was further purified by pre-HPLC using a reversed-phase C18 silica gel column with the mobile phase consisted of acetonitrile and H_2O, and collected the fractions. All the fractions were applied on PM H^+-ATPase activity test and three fractions 1, 2 and 3 showed activity in stimulating PM H^+-ATPase activity.

Black lines below the fractions marked the fractions that activate PM H^+-ATPase activity. DCM, dichloromethane; MeOH, methanol; PE, petroleum ether; pre-HPLC, preparative high performance liquid chromatography; silica gel, column chromatography was performed over silica gel; LH-20, column chromatography was performed over Sephadex LH-20.

Supplemental Figure 2

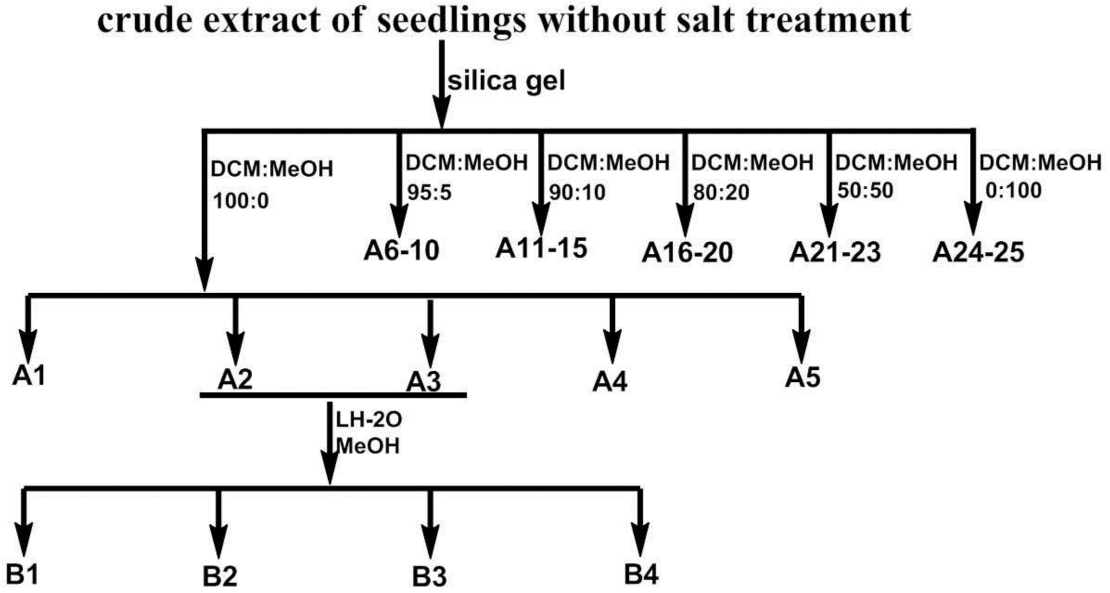

Supplemental Figure 2. The bio-guided isolation procedure of the seedlings without salt treatment.

The MeOH extract (2.20 g) was subjected to a silica gel normal-phase column, eluting with a stepwise gradient solvent of DCM-MeOH (100:0, 95:5, 90:10, 80:20, 50:50, 0:100) to give 25 fractions (FrA1-FrA25). All fractions were applied on PM H^+-ATPase activity test and no fraction showed activity in stimulating PM H^+-ATPase activity. FrA2-FrA3 were further combined and subjected to a Sephadex LH-20 column eluting with MeOH to give four fractions (FrB1-FrB4). All these four fractions were applied on PM H^+-ATPase activity test and no fraction showed activity in stimulating PM H^+-ATPase activity.

Black lines below the fractions marked the fractions that activate PM H^+-ATPase activity in seedlings with salt treatment, however, no activity in seedlings without salt treatment.

DCM, dichloromethane; MeOH, methanol; silica gel, column chromatography was performed over silica gel; LH-20, column chromatography was performed over Sephadex LH-20.

Supplemental Figure 3

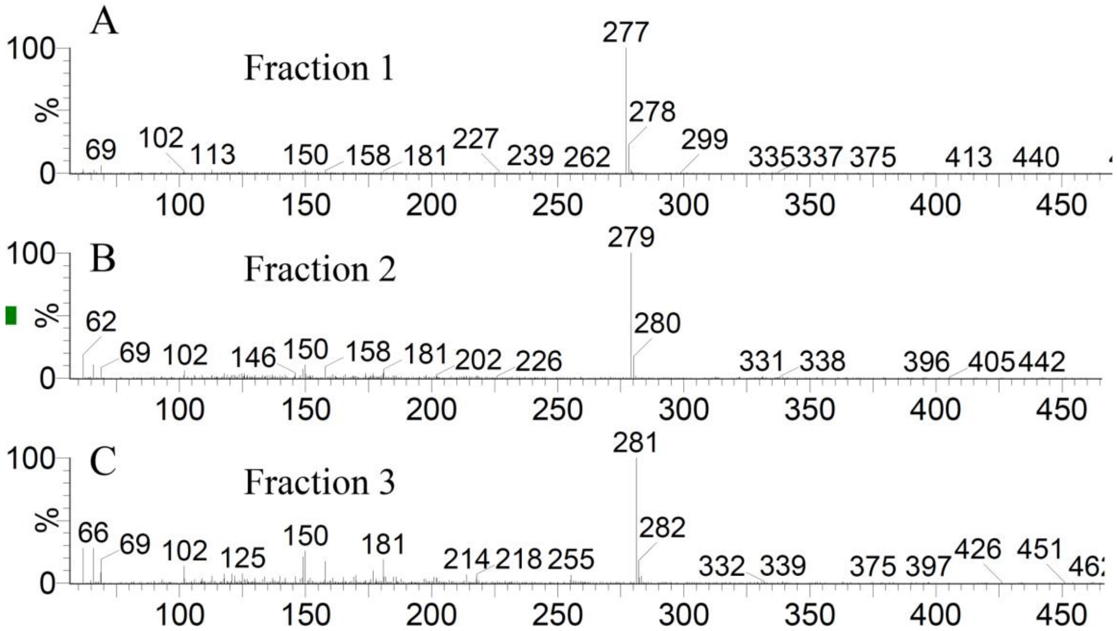

Supplemental Figure 3. The LRESIMS spectrum of compounds in fractions 1, 2 and 3.

(A) LRESIMS (ESI^-) of compound in fraction 1 in the process of preparation.

(B) LRESIMS (ESI^-) of compound in fraction 2 in the process of preparation.

(C) LRESIMS (ESI^-) of compound in fraction 3 in the process of preparation.

Supplemental Figure 4

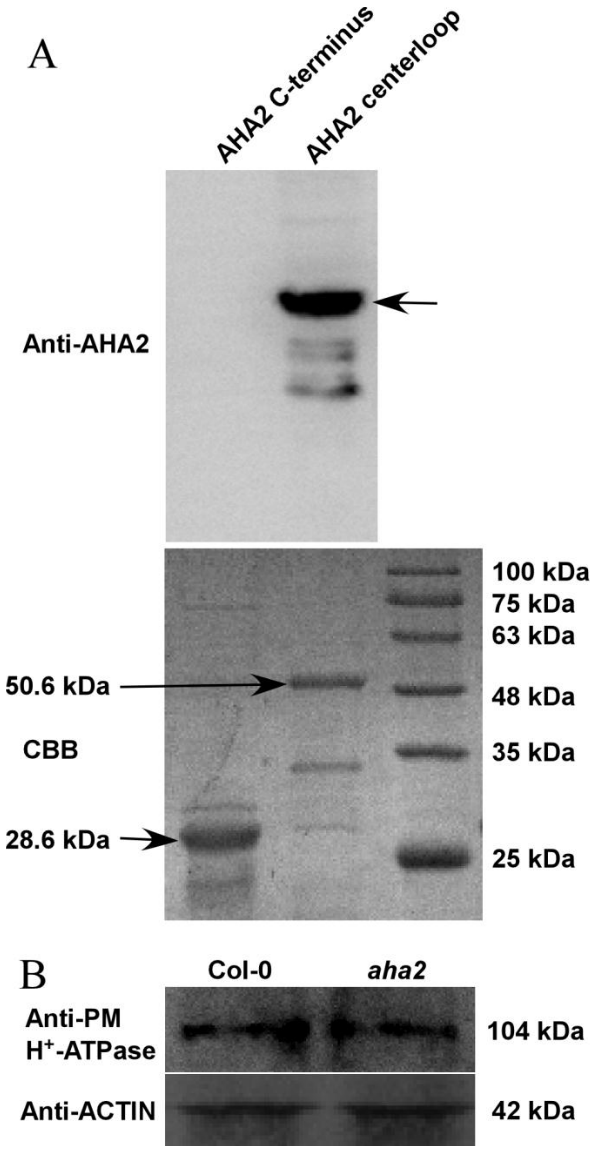

Supplemental Figure 4 Availability of the AHA2 antibody.

(A) Upper panel, AHA2 centerloop recombination protein and AHA2 C-terminus recombination protein were analyzed by Western blotting using the anti-PM H^+-ATPase antibody against the centerloop peptide; lower panel, CBB (Coomassie Brilliant Blue staining). (B) Upper panel, proteins were extracted from Col-0 or *aha2* mutant seedlings and analyzed by Western blotting using the anti-PM H^+-ATPase antibodies; lower panel, proteins were extracted from Col-0 or *aha2* mutant and analyzed by Western blotting using the anti-ACTIN antibody as an internal control.

Supplemental Figure 5

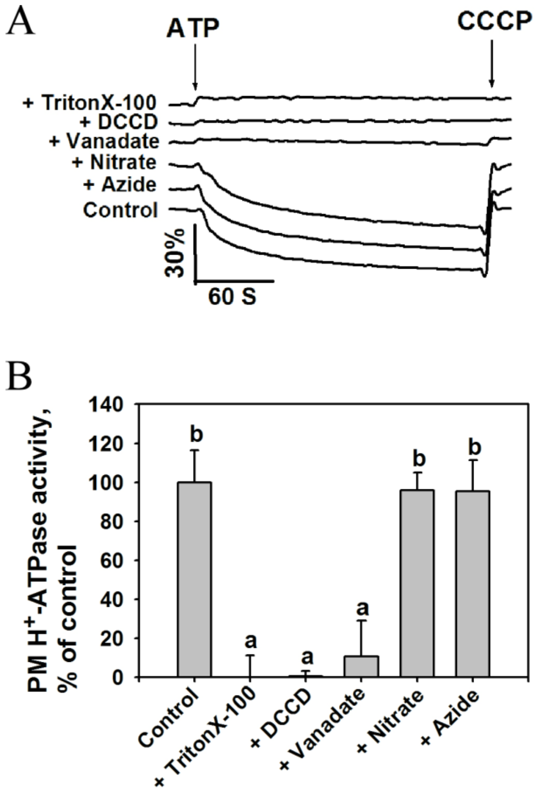

Supplemental Figure 5. Proton transport competency of the isolated vesicles. Plasma membrane vesicles were isolated from leaves of Col-0. PM H^+-ATPase activity was measured in vesicles in the presence of different inhibitors as follows:

(A) PM H^+-ATPase activity was measured in vesicles with vanadate (1 mM, a P-type ATPase inhibitor), nitrate (50 mM, a V-type ATPase inhibitor), Triton X-100 (0.1%, a detergent that causes the membranes to be leaky), N,N’-dicyclohexylcarbodiimide (DCCD, 10 µM, a H^+ channel inhibitor) and without inhibitor (control).

(B) Comparison of PM H^+-ATPase activity in panel A. The data in panels B represent means ± standard deviation (SD) of five replicates. A Student’s t-test was used to analyze statistical significance; significant differences ($P \le 0.05$) in panels B are indicated by different lowercase letters.

Supplemental Figure 6

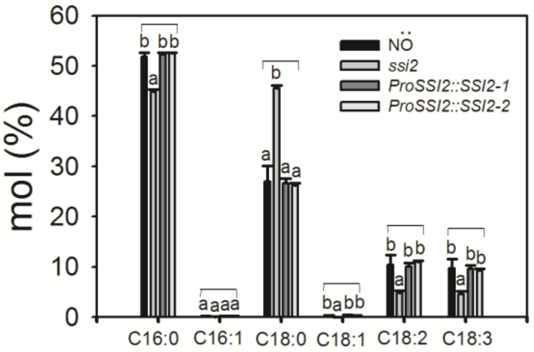

Supplemental Figure 6. Fatty acid analyses of NÖ, *ssi2*, and *ProSSI2::SSI2-1* and *ProSSI2::SSI2-2* seedlings.

Arabidopsis seeds were sterilized and sown in the solid MS medium plus 25 g/L sucrose and grown under continuous white light (light intensity of 50 µmol m^{-2} s^{-1}) at 23°C for 7 days, then the seedlings were transferred to soil for 2-weeks under 16-h-light (22°C)/8-h-dark (20°C) cycle and leaves were collected for the fatty acids analyses.

Three independent experiments displayed similar results. The data represents means ± standard deviation (SD). A Student's t-test was used to analyze statistical significance; significant differences (P$\leqslant$0.05) are indicated by different lowercase letters.

Supplemental Figure 7

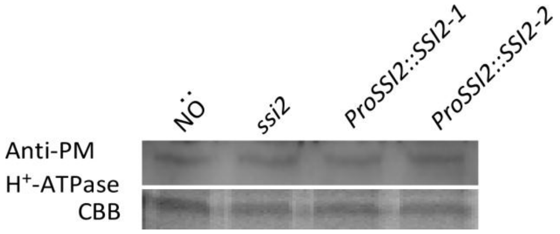

Supplemental Figure 7. PM H^+-ATPase protein levels in NÖ, *ssi2*, *ProSSI2::SSI2-1*, and *ProSSI2::SSI2-2* seedlings.

Upper panel, plasma membrane vesicles were isolated from NÖ, *ssi2*, *ProSSI2::SSI2-1*, and *ProSSI2::SSI2-2* seedlings and PM H^+-ATPase protein levels in the samples analyzed by Western blotting using the anti-PM H^+-ATPase antibody; lower panel, CBB (Coomassie Brilliant Blue staining).

Supplemental Figure 8

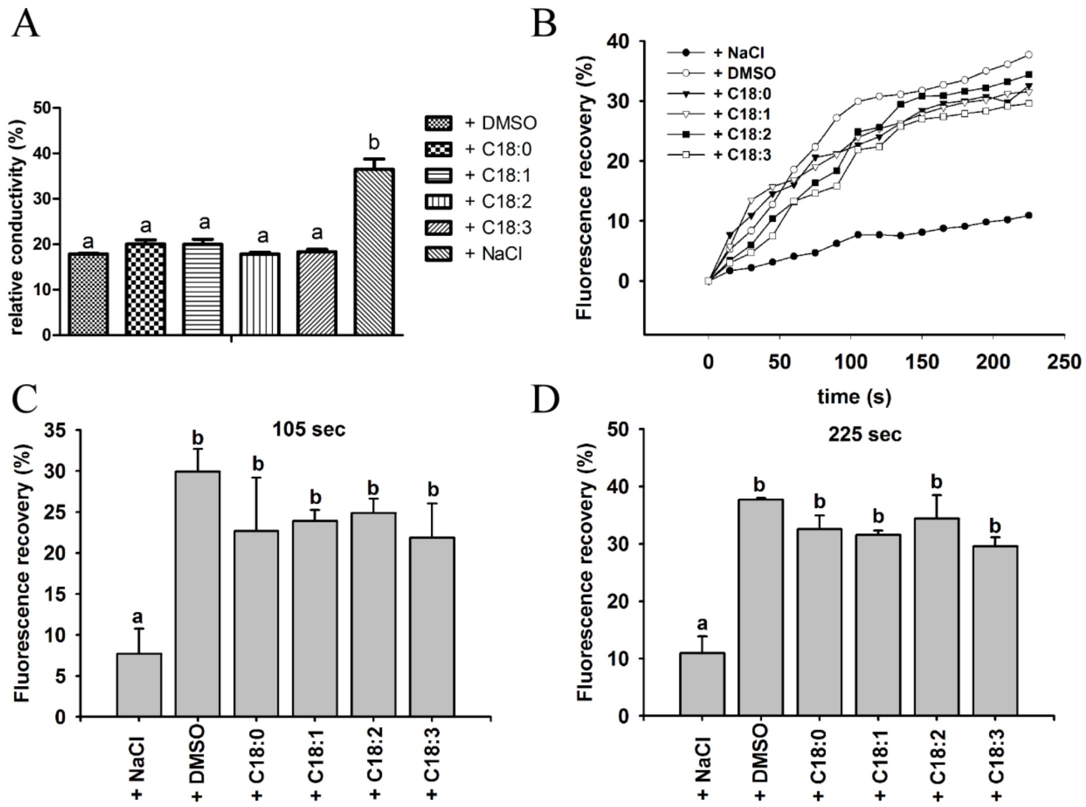

Supplemental Figure 8. Exogenous application of C18:1, C18:2 or C18:3 does not influence membrane conductivity and membrane fluidity.

(A) Relative membrane conductivity was measured with conductivity meter. Leaves were treated with 100 µM of C18:0, C18:1, C18:2, C18:3, solvent control (0.1% DMSO), and 150 mM NaCl for 12 hours, respectively.

(B) The fluorescence recovery rate, an index of membrane fluidity, was measured by FARP technology. Five-day-old *Arabidopsis* roots were treated with 100 µM of C18:1, C18:2, C18:3, solvent control (0.1% DMSO) and 500 mM NaCl for 10 mins respectively, and then stained with FM 4-64 for 10 mins. A plasma membrane region was selected and the fluorescence intensity of the region was measured in pre- and post-bleach images. The figures were then analyzed using Image J software. The fluorescence recovery rate is the percentage of post-bleach fluorescence intensity versus pre-bleach fluorescence intensity.

(C) The fluorescence recovery rate at 105 sec from panel B.

(D) The fluorescence recovery rate at 225 sec from panel B.

The data in panels A, C and D represent means ± standard deviation (SD) of five replicates. A Student's t-test was used to analyze statistical significance; significant differences ($P \leqslant 0.05$) in panels A, C and D are indicated by different lowercase letters.

Supplemental Figure 9

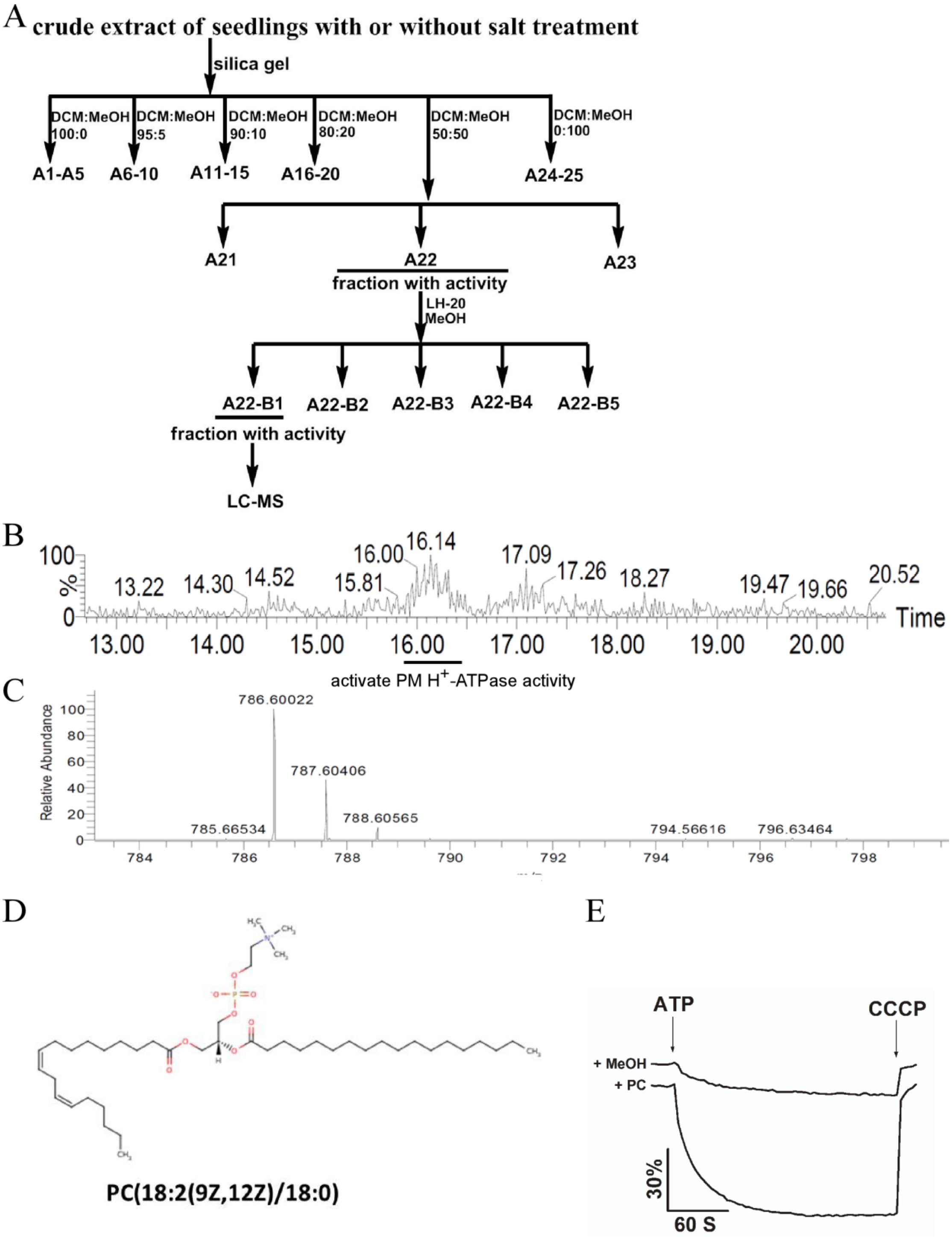

Supplemental Figure 9 The fraction containing PC activates PM H^+-ATPase activity.

(A) The bio-guided isolation procedure of active fraction containing PC. The MeOH extract was subjected to a silica gel normal-phase column, eluting with a stepwise gradient solvent of DCM-MeOH (100:0, 95:5, 90:10, 80:20, 50:50, 0:100) to give 25 fractions (FrA1-FrA25). All fractions were applied on PM H^+-ATPase activity test. FrA22 with activity was further subjected to a Sephadex LH-20 column eluting with MeOH to give five fractions (FrA22-B1-FrA22-B5). All these five fractions were applied on PM H^+-ATPase activity test and FrA22-B1 showed activity in

stimulating PM H^+-ATPase activity. FrA22-B1 was further applied on LC-MS analysis and separation.

(B) LC-MS (ESI^+) preparation of fraction A22-B1. Small amount (0.5 mg) of fraction A22-B1 was applied on LC-MS preparation using analysis column (4.6 mm x 150 mm) and collected using Eppendorf tubes. Repeated preparation was applied and combined. All fractions were solvent-evaporated using a concentrator (Eppendorf Concentrator Plus) and applied on PM H^+-ATPase activity test. A fraction in the retention time of 16.14 min showed activity in stimulating PM H^+-ATPase activity.

(C) HRESIMS (ESI^+) information of fraction in 16.14 min.

(D) Structure of 786.60022 $[M+H]^+$ from metlin (https://metlin.scripps.edu) and PC was identified (calcd 786.6013).

(E) PC activates PM H^+-ATPase activity. PM H^+-ATPase activities measured in vesicles in the presence of 100 µM PC or solvent control (0.1% MeOH, V/V). Plasma membrane vesicles were isolated from 5-week-old *Arabidopsis* (ecotype Col-0) seedlings.

Supplemental Figure 10

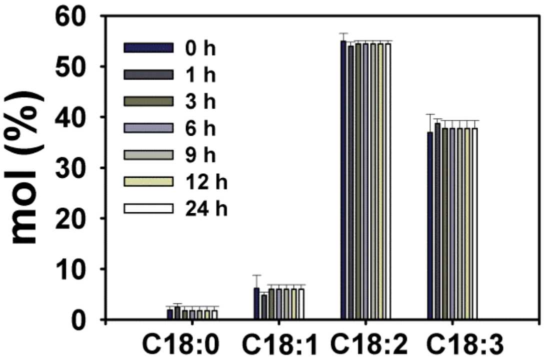

Supplemental Figure 10. Total fatty acid analyses of col-0 under salt stress. *Arabidopsis* seeds Col-0 were sterilized and sown in the liquid 1/2 MS medium plus 10 g/L sucrose and grown under dark for 20 days and then treated with 150 mM NaCl for different hours indicated above, and the roots were collected for the fatty acids analysis.

Three independent experiments displayed similar results. The data represents means ± standard deviation (SD). A Student's t-test was used to analyze statistical significance; significant differences ($P \leqslant 0.05$) are indicated by different lowercase letters.

Supplemental Figure 11

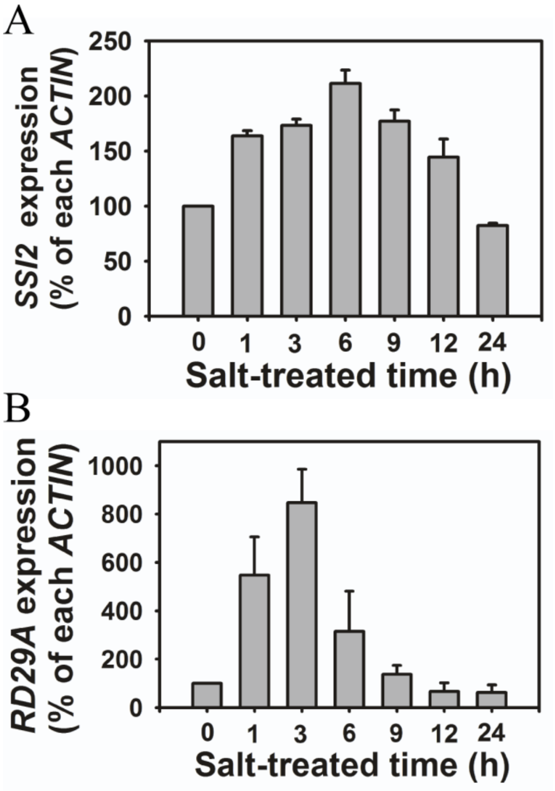

Supplemental Figure 11 Expression of *SSI2* after salt treatment.

(A) Quantitative real time RT-PCR assay for detection of *SSI2* expression after salt treatment.

(B) Quantitative real time RT-PCR assay for detection of *RD29A* expression after salt treatment.

Total RNA was extracted with RNAVzol kit (Vigorous) from 10-day-old seedlings treated with 100 mM NaCl for 0, 1, 3, 6, 9, 12, 24 h respectively. The samples were treated with RNase-free DNase to eliminate genomic DNA, and they were used for reverse transcription assay. Then the cDNAs were used for quantitative real-time PCR assay. *RD29A* served as a marker gene in the response of salt stress. *ACTIN* served as an internal control.

Supplemental Table 1

Addition	H^+-ATPase activity (μM Pi mg^{-1} protein min^{-1}) and inhibit effect (%)
	Upper phase
Control (Without inhibitor)	1.872 ± 0.120 (0)
+ NaN_3 (1 mM)	1.798 ± 0.110 (3.98)
+ $NaNO_3$ (50 mM)	1.848 ± 0.166 (1.26)
+ $(NH_4)_6Mo_7O_{24}$ (0.1 mM)	1.758 ± 0.113(5.11)
+ Na_3VO_4（1 mM)	0.189 ± 0.145(89.95)

Supplemental Table 1. Plasma membrane purity determination.

Plasma membrane vesicles were isolated from leaves of Col-0. Sodium azide (NaN_3, 1 mM), sodium nitrate ($NaNO_3$, 50 mM), sodium molybdate ($(NH_4)_2MO_4$, 0.1 mM) or sodium ortho vanadate (Na_3VO_4, 1 mM) were added to assays to assess the percent of ATPase hydrolytic activity originating from mitochondrial membranes (azide) , vacuolar membranes (nitrate) , soluble phosphatases (molybdate) or plasma membranes (vanadate). Sodium azide- and sodium nitrate-sensitive ATPase activities were measured at pH 7.5, ortho vanadate-sensitive ATPase activity was measured at pH 6.5. Hydrolytic activities of inhibitor-dependent changes were calculated based on the activity of a boiled-membrane control at each assay pH. The data represent means of five replicates.

Supplemental Table 2

Position	**1**	**2**
	δ_H (J Hz)	δ_H (J Hz)
2	2.35, t (7.2)	2.35, (7.2)
3	1.65 [a], m	1.65 [a], m
4	1.31 [a], m	1.31 [a], m
5	1.31 [a], m	1.31 [a], m
6	1.31 [a], m	1.31 [a], m
7	1.31 [a], m	1.31 [a], m
8	2.05 [a], m	2.05 [a], m
9	5.36 [a], m	5.36 [a], m
10	5.36 [a], m	5.36 [a], m
11	2.81, t (6.5, 5.6)	2.77, dd (6.7 6.2)
12	5.36 [a], m	5.36 [a], m
13	5.36 [a], m	5.36 [a], m
14	2.81, t (6.5, 5.6)	2.05 [a], m
15	5.36 [a], m	1.31 [a], m
16	5.36 [a], m	1.31 [a], m
17	2.05 [a], m	1.31 [a], m
18	0.98 (J = 7.6)	0.89, t (6.8)

Supplemental Table 2. ^{1}H NMR spectral data of compounds in fractions 1 and 2 ($CDCl_3$, 400 MHz).

a Multiplicities were unclear due to overlapping signals.

Supplemental Table 3

Addition	H^+-ATPase activity (µM Pi mg^{-1} protein min^{-1}) and relative to control (%)
Control (+ 0.1% (V/V) DMSO)	1.951 ± 0.084 (100)
+ 100 µM C18:0	1.987 ± 0.114 (101.82)
+ 100 µM C18:1	2.740 ± 0.126 (140.45)
+ 100 µM C18:2	2.785 ± 0.130 (142.75)
+ 100 µM C18:3	2.895 ± 0.160 (148.38)

Table 3 Fatty acid on plasma membrane H^+-ATPase hydrolytic activity

Plasma membrane vesicles were isolated from leaves of Col-0. H^+-ATPase hydrolytic activities were measured at pH 6.5. The vesicles were incubated in the assay buffer containing 0.1% (V/V) DMSO (control), 100 µM of C18:0, C18:1, C18:2 or C18:3 (C18:0, C18:1, C18:2 or C18:3 was dissolved in DMSO with a stock concentration of 100 mM). Hydrolytic activities were calculated by the liberation of Pi from ATP (µM Pi per mg protein per min). The data represent means of five replicates.

Supplemental Table 4

Samples	H^+-ATPase activity (µM Pi mg^{-1} protein min^{-1}) and relative to control (%)
NÖ	2.909 ± 0.115 (100)
ssi2	2.523 ± 0.171 (86.73)
ProSSI2::SSI2-1	2.908 ± 0.165 (99.97)
ProSSI2::SSI2-2	2.930 ± 0.167 (100.72)

Table 4 H^+-ATPase hydrolytic activity of NÖ, *ssi2*, *ProSSI2::SSI2-1* and *ProSSI2::SSI2-2* seedlings Plasma membrane vesicles were isolated from leaves of NÖ, *ssi2, ProSSI2::SSI2-1* and *ProSSI2::SSI2-2* treated with 250 mM NaCl for 3 days before harvested. H^+-ATPase hydrolytic activities were measured at pH 6.5. Hydrolytic activities were calculated by the liberation of Pi from ATP (µM Pi per mg protein per min). The data represent means of five replicates.
